# Analysis of influencing factors and establishment of prediction model for successful vaginal delivery after cesarean section

**DOI:** 10.3389/fgwh.2025.1447569

**Published:** 2025-02-24

**Authors:** Hongxia Zhang, Lin Yu, Songquan Wei, Guiming Li

**Affiliations:** ^1^ Department of the Third Affifiliated Hospital, Guangzhou Medical University, Guangzhou, Guangdong, China; ^2^ Department of Obstetrics and Gynecology, Third Affiliated Hospital of Guangzhou Medical University, Guangzhou, Guangdong, China

**Keywords:** vaginal birth after cesarean, cesarean section, pregnancy complications, neonatal outcomes, prediction model

## Abstract

**Objective:**

To explore the influencing factors of vaginal delivery after cesarean section, establish a predictive model, and identify potential factors for perinatal complications.

**Materials and methods:**

This is a retrospective analysis of women who attempted a trial of labor after cesarean section(TOLAC) at the Third Affiliated Hospital of Guangzhou Medical University and subsequently gave birth in this hospital between 31 December 31 2017 and December 2023. Associations between maternal characteristics and success of TOLAC were assessed using univariate and logistic regression. A predictive model was developed and performance was assessed using the acceptor-operator curve (ROC).

**Results:**

A total of 10,277 pregnant women with a history of previous cesarean section were identified during the observation period, 1,065 attempted TOLAC, which 839 were successful vaginal birth after cesarean (VBAC) and 226 failed vaginal trials. We have developed and validated a simple nomogram prediction model based on common antenatal predictors, which are independently associated with successful TOLAC, including maternal age, height, cervical Bishop score, estimated fetal weight, and use of oxytocin and artificial rupture of membranes to induce labor. The prediction model has been established and verified, and the model demonstrates good prediction efficiency, with an area under the ROC curve of 83.1%. Compared with the TOLAC-failure group and the ERCD group, the VBAC group had the lowest amount of bleeding in intrapartum and 24 h after delivery, puerperal infection, and uterine rupture. Nevertheless, the prevalence of placental abruption and the incidence of neonatal neonatal intensive care unit were higher in this cohort.

**Conclusion:**

TOLAC is an important public health strategy in China. The results of our study can be used to improve counselling, reduce decision-making conflicts and increase the success rate of trials of vaginal delivery, ultimately improving the prognosis for mother and baby, by providing case-specific possibilities for counselling and management of women with a history of caesarean section and according to the characteristics of each pregnancy.

## Introduction

In 2010, the World Health Organization (WHO) observed that the incidence of caesarean sections in China was considerably higher than that observed in other Asian countries (1). A study has indicated that by 2020, the caesarean section rate in secondary and higher hospitals in China will be as high as 60% (2). The main factor for pregnant women to choose cesarean section delivery is uterine scar. However, selective repeat cesarean section (ERCD) after cesarean section has been shown to increase the risk of perinatal complications. Therefore, encouraging vaginal delivery after cesarean section and improving the success rate of vaginal delivery after cesarean section is the main challenge (3–5).

In order to avoid repeated cesarean section, China issued an expert consensus on VBAC management in 2016 (6), aimed at supporting medical institutions with conditions to provide TOLAC opportunities to suitable pregnant women. Some medical institutions in China have also gradually tried TOLAC, but due to the risks of emergency cesarean section (TOLAC) failure converted to cesarean section), placental abruption, bleeding, blood transfusion, uterine rupture, and endometritis in TOLAC, the enthusiasm of general medical institutions for TOLAC is not high (7–9). Successful vaginal delivery avoids major abdominal surgery, while the incidence of bleeding, thrombosis, pelvic adhesions and infections is lower, and the length of hospital stay and recovery period are also shorter than those of women with ERCD (10–14). In addition, for women who plan to have another pregnancy in the future, VBAC may reduce the risk of complications associated with multiple cesarean sections (e.g., hysterectomy, intestinal or bladder injury, placental location abnormalities such as placenta previa, and placenta hyperplasia) (15–17). In the tense doctor-patient relationship in China, medical staff need to intervene effectively to avoid medical disputes caused by the failure of TOLAC. According to the China Health Statistical Yearbook (2023), in 2022, medical institutions nationwide reported a total of about 120,000 medical disputes, of which tertiary hospitals accounted for more than 60% (18). Incomplete statistics show that obstetric disputes account for 40%–50% of all medical disputes (19). With the promulgation of laws and regulations such as the Civil Code and the Regulations on the Prevention and Handling of Medical Disputes, the handling of medical disputes has become more diverse. This legal risk has caused medical professionals to avoid high-risk surgeries. For example, concerns about complications such as uterine rupture and fetal distress, which may lead to litigation, often hinder the promotion of TOLAC.

According to the inclusion criteria described in the clinical guidelines (6), this retrospective study aimed to identify factors that influence the success rate of TOLAC, and develop a predictive model to guide effective implementation of clinical guidelines.

## Material and methods

### Data source

This retrospective cohort study included women with a single previous caesarean section and delivered their current pregnancy at the Third Affiliated Hospital of Guangzhou Medical University, between 31 December 2017 and 31 December 2023. The hospital is a public tertiary care facility. Ethical approval for the study was granted by the hospital's human research ethics committee for the purpose of reviewing the medical records.

**Figure 1:**
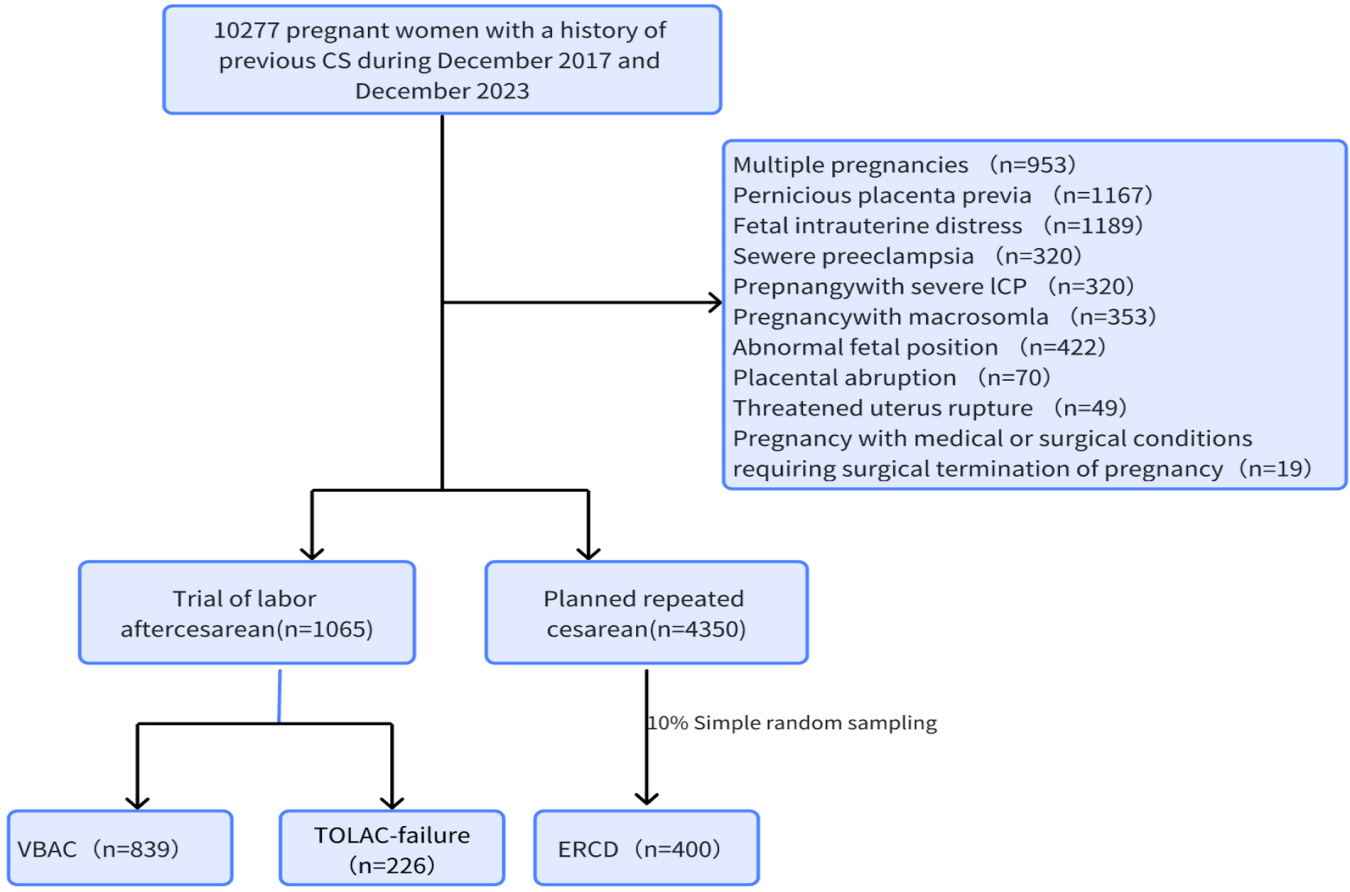
Study cohort and enrollment process.

The inclusion criteria are as follows: (1) The desire of the pregnant woman to give birth vaginally is a necessary condition for TOLAC, and this is a singleton pregnancy; (2) A history of a previous transverse incision caesarean section at the lower segment of the uterus is present, and the previous caesarean section was successful, with no extension of the incision, recovery as expected, and no late postpartum bleeding, postpartum infection, etc. No other surgical scars in the uterus are present, except for the caesarean section incision; (3) The fetus is in a cephalic position; (4) Absence of any previous indications for caesarean section (e.g., abnormal fetal position, breech presentation, transverse presentation), placenta previa, placenta accreta, severe pre-eclampsia, placental abruption, multiple gestation, presence of umbilical cord, prolapse, and occult prolapse were not identified as indications for caesarean section; (5) The interval between two deliveries was ≥18 months; (6) The EFV was less than 4,000 g. The exclusion criteria are as follows include: (1) Patients who have had undergone two or more previous caesarean sections, or those who have undergone classical caesarean sections, longitudinal incisions of the lower uterine segment, T-shaped incisions, or previous uterine ruptures; (2) Indications for previous caesarean sections; (3) The EFV was greater than or equal to 4,000 g; (4) A history of uterine rupture or a history of myomectomy with uterine cavity penetration; (5) Previous caesarean section with complications related to the uterine incision; (6) Previous caesarean section with complications related to the uterine incision; (7) Surgical or medical complications or obstetric complications that make vaginal delivery inappropriate, such as heart failure, severe liver and kidney disease, and severe pre-eclampsia with organ dysfunction. The process used to form the study groups is shown as a flow chart in Figure 1.

### Data collection

Success rate of TOLAC was the outcome variable, whereas socio-demographic factors (age, height, pre-pregnancy weight, pre-delivery weight, pre-pregnancy weight gain), present and past obstetric history (parity, gravidity, inter-delivery interval, vaginal delivery history, cervical Bishop score, natural labor, premature rupture of membrane, artificial rupture of membrane, estimated fetal weight) were independent variables. Perinatal complications and neonatal outcomes (such as intrapartum and 24-hour postpartum blood loss, puerperal infection, uterine rupture, placental abruption, neonatal asphyxia, and neonatal admission to the neonatal intensive care unit (NICU) were compared between VBAC and TOLAC-failure, VBAC and ERCD.

Indicator definitions: (1) Postpartum hemorrhage (PPH): estimated blood loss of more than 500 ml during vaginal delivery and more than 1,000 ml during cesarean delivery (20, 21); (2) The diagnostic criteria for neonatal asphyxia:Five minutes Apgar scores <7 (22). (3) Determination of the cervical Bishop score at the time of admission: The cervical Bishop score was determined on admission by midwives or clinicians with a minimum of five years of experience. The score was based on the following criteria: uterine opening, cervical regression, presentation location, cervical stiffness, and uterine opening position. The total score ranged from 0 to 13.

### Statistical analysis

**Figure 2:**
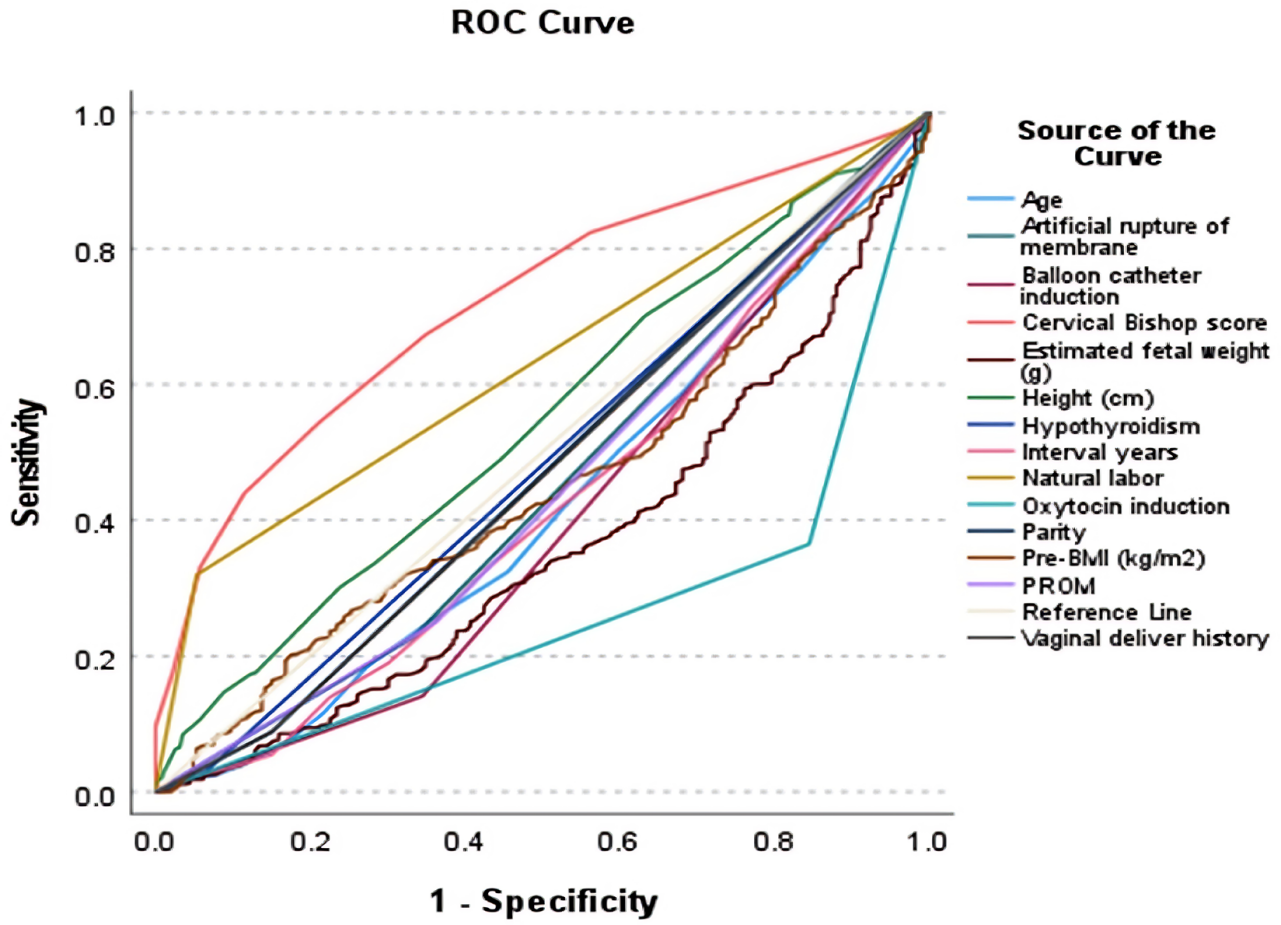
ROC curve of indicators used to predict VBAC.

**Figure 3:**
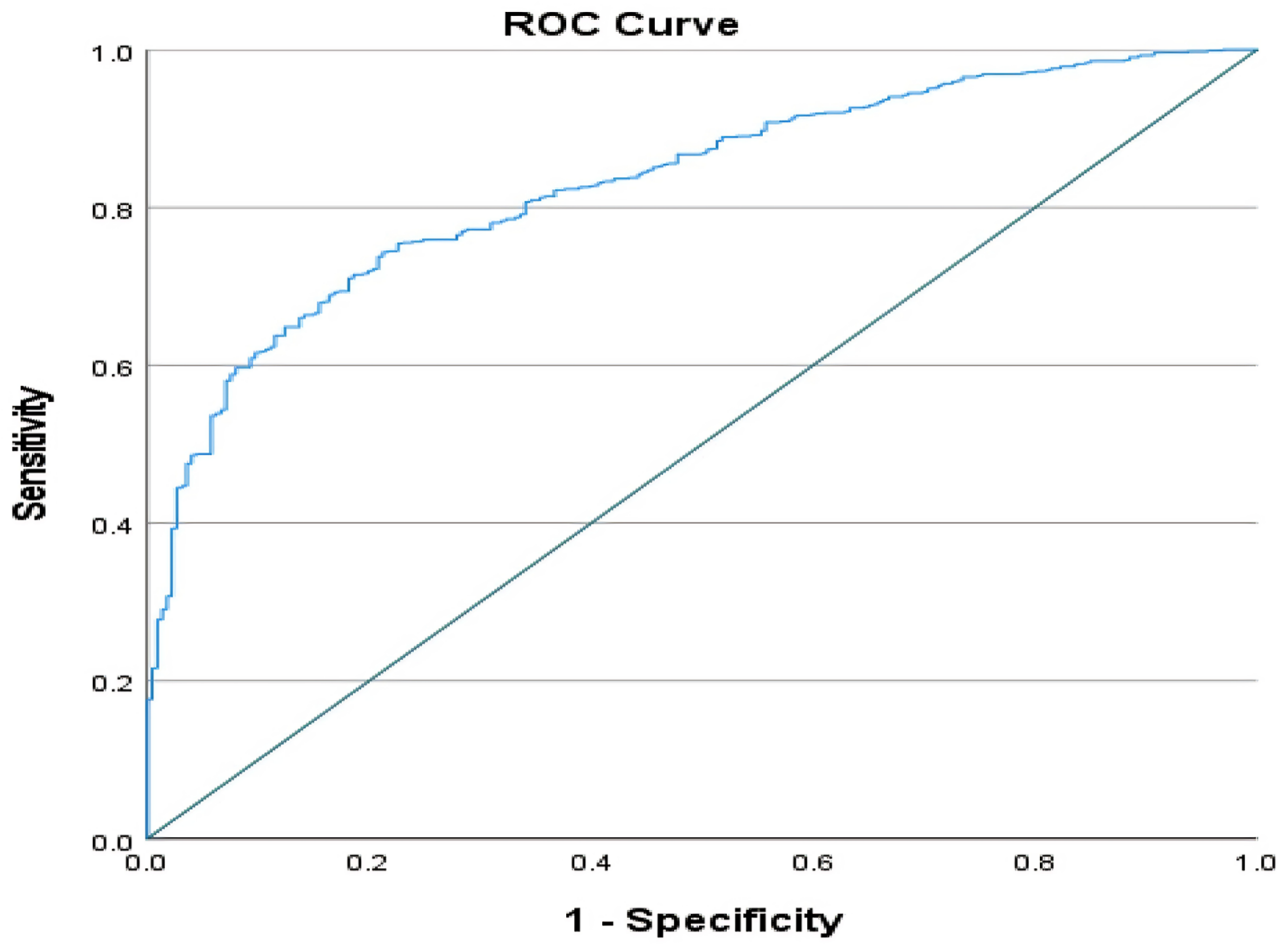
The ROC curve of the VBAC prediction model.

The statistics were analyzed using SPSS version 25.0. The quantitative data was examined for normal distribution, and normally distributed variables were represented as the mean ± SD, and a t-test was used to analyse them. Non-parametric continuous variables were represented as the median and interquartile range, and the Mann–Whitney *U* test was applied to analyse them. The receiver operating characteristic (ROC) curve was used to compare the contribution of each factor to predict VBAC (Figure 2). The highest predictive value was determined using the Youden index. The impact of each contributing factor is quantified by the odds ratio and its associated confidence interval (OR and 95% CI). A multivariate logistic regression model was constructed to predict the probability of a successful VBAC, and a regression equation was derived. The model demonstrated a high degree of predictive accuracy, with an area under the ROC curve of 83.1%. (Figure 3) All tests were conducted at a 0.05 significance level.

## Results

During the observation period, a total of 10,277 pregnant women with a history of cesarean sections were found. Multiple pregnancies (9.2%), placenta previa with or without implantation (11.4%), severe pre-eclampsia (3.1%), severe ICP in pregnancy (3.1%), macrosomia in pregnancy (3.4%), malposition (breech, transverse) (4.1%), internal and external diseases in pregnancy (0.2%), and intrauterine distress (10.6%), placental abruption (0.7%), threatened uterine rupture (0.5%) were excluded. There were 1,065 (19.67%, 1,065/5,415) pregnant women who chose TOLAC and 4,350 (80.33%, 4,350/5,415) pregnant women who chose ERCD, of which 839 cases were VBAC. The success rate of vaginal trial labor was 79%. There were 226 cases of TOLAC failure. General and clinical characteristics of women who chose TOLAC are shown in Table 1. Analysis of the causes revealed that the most common reason for a cesarean section was non-reassurance fetal heart rate pattern or non-reassuring fetal heart rate patterns (NRFHRP)in 117 cases (51.77%, 117/226), followed by pregnant women afraid of the pain during labour and refused to continue vaginal trial labour in 51 cases (22.57%, 51/226).

**Table 1:** Comparison of the VBAC group and the TOLAC-failure group.

	VBAC (*n* = 839)	TOLAC-failure (*n* = 226)	*P*
Age (years)^a^	32.55 ± 4.19	33.85 ± 4.40	0.000
Height (cm)^a^	159.09 ± 5.05	158.28 ± 4.64	0.029
Pre-BMI (kg/m^2^)^a^	21.65 ± 3.07	22.20 ± 3.12	0.019
<18	72 (8.58)	7 (3.10)	
18–24	576 (68.65)	171 (75.66)	
24–30	184 (21.93)	42 (18.58)	
≥30	7 (0.83)	6 (2.66)	
Gravidity^b^	2 (2, 3)	2 (2,3)	0.9945
Parity^b^	1 (1,1)	1 (1,1)	0.045
Vaginal deliver history^c^	74 (8.82)	34 (15.04)	0.006
Weight gain (kg)^a^	12.75 ± 4.37	13.16 ± 4.33	0.211
Interval months (years)^a^	5.66 ± 3.22	6.67 ± 3.69	0.000
BMI during the delivery^a^	26.90 ± 7.61	27.44 ± 3.35	0.292
<18	3 (0.36)	0 (0.00)	
18–24	178 (21.22)	32 (14.16)	
24–30	536 (63.89)	150 (66.37)	
≥30	122 (14.54)	44 (19.47)	
Cervical bishop score^a^	5.16 ± 2.69	3.19 ± 1.72	0.000
Induction of labour (IOL)			
Oxytocin induction^c^	306 (36.47)	35 (15.49)	0.000
Balloon catheter induction^c^	118 (14.06)	78 (34.51)	0.000
Artificial rupture of membrane induction^c^	190 (22.65)	75 (33.19)	0.001
Natural labor^c^	268 (31.94)	12 (5.31)	0.000
PROM^c^	210 (25.03)	82 (36.28)	0.001
Pregnancy complications			
GDM^c^	177 (21.10)	46 (20.35)	0.444
Hypertensive disease^c^	39 (4.65)	8 (3.54)	0.304
Hypothyroidism^c^	26 (3.10)	15 (6.64)	0.016
Hyperthyroidism^c^	13 (1.55)	2 (0.88)	0.307
ICP^c^	7 (0.83)	2 (0.88)	0.641
Estimated fetal weight(g)^a^	2,843.29 ± 592.32	3,100.88 ± 495.24	0.000
<3,500 g^a^	764 (91.06)	186 (82.30)	
≥3,500 g^a^	75(8.94)	40(17.70)	

Index, PROM, premature rupture of fetal membranes; GDM, gestational diabetes mellitus; ICP, intrahepatic cholestasis during pregnancy.

^a^ Paired *t*-test with two independent samples was used.

^b^ Non-parametric Mann–Whitney U test was used.

^c^ Chi-sqare test was used.

### Univariate analysis of VBAC

Single factor analysis showed that maternal age, height, pre-pregnancy BMI, parity, vaginal delivery history, inter-delivery interval, cervical Bishop score, labor induction method (oxytocin, balloon catheter, artificial rupture of membranes), natural labor, premature rupture of membranes, pregnancy complications (hyperthyroidism), and estimated fetal weight were correlated with TOLAC outcome (*P* < 0.05). The TOLAC outcome was not correlated with BMI, pregnancy size, pregnancy weight gain, or pregnancy complications (GDM, hypothyroidism, hypertensive disease during pregnancy, ICP) (*P* > 0.05) (Table 2). Based on the results of univariate analysis, we used ROC curves were employed to compare the contribution of each factor to the prediction of VBAC. This is illustrated in Figure 2. The Jorden index was employed to ascertain the most predictive value, with the cervical Bishop score being identified as the most predictive value (AUC = 0.721), followed by the Natural labor (AUC = 0.633) Table 3.

**Table 2:** Comparison of the area under the ROC curve of each indicator and their value in VBAC prediction.

	AUC	95%CI		
		Lower limit	Upper limit	*P*
Age	0.419	0.376	0.461	0.000
Height (cm)	0.544	0.502	0.585	0.040
Pre-BMI (kg/m^2^)	0.451	0.411	0.491	0.017
Interval years	0.423	0.380	0.466	0.000
Estimated fetal weight (g)	0.360	0.320	0.400	0.000
Cervical Bishop score	0.721	0.687	0.755	0.000
Vaginal deliver history	0.469	0.425	0.512	0.161
Oxytocin induction	0.260	0.226	0.294	0.000
Balloon catheter induction	0.398	0.354	0.442	0.000
Artificial rupture of membrane	0.447	0.404	0.491	0.017
Natural labor	0.633	0.597	0.670	0.000
PROM	0.444	0.401	0.487	0.011
Hypothyroidism	0.482	0.439	0.525	0.422
Parity	0.473	0.429	0.516	0.221

**Table 3:** Analysis of independent risk factors of VBAC through multivariate logistic regression.

	*B*	Standard error	Wald *χ*^2^	*P*	OR	95% CI
Age	−0.054	0.024	4.809	0.028	0.948	0.904–0.994
Height	0.076	0.019	16.326	0.000	1.079	1.040–1.119
Cervical bishop score	0.210	0.056	13.883	0.000	1.233	1.105–1.377
Estimated fetal weight	−0.001	0.000	20.762	0.000	0.999	0.999–0.999
Oxytocin induction	−1.613	0.237	46.333	0.000	0.199	0.125–0.317
Artificial rupture of membrane	−0.523	0.204	6.579	0.010	0.593	0.398–0.884
Constant	−5.061	3.055	2.744	0.098	0.006	

### Predictors of TOLAC success

**Figure 4:**
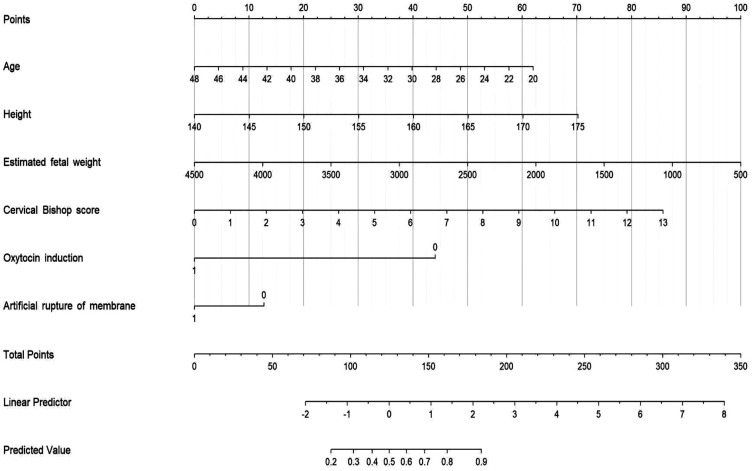
Predictive graphic nomogram for probability of vaginal birth after caesarean delivery.

Multivariate, stepwise and backward logistic regression analyses were performed on the variables screened by univariate analysis as being associated with the TOLAC outcome. In the end, only 6 indicators were used as independent predictors of TOLAC, and a regression model was established. The regression was as follows: the predicted probability of TOLAC = −5.061−0.054* Age + 0.076* Height (cm) + 0.210* Cervical Bishop score −0.001* Induced oxytocin (yes = 1, no = 0)−1.163* Estimated fetal weight (g) −0.542* Artificial rupture of membrane (yes = 1, no = 0). In addition, we have developed and validated a simple nomogram prediction model based on common antenatal predictors, which are independently associated with successful TOLAC, including maternal age, height,cervical Bishop score,estimated fetal weight,use of oxytocin and artificial rupture of membranes to induce labor. The nomogram transforms each risk predictor into a 0–100 scale, proportional to the calculated adjusted log odds. Subsequently, the total score, calculated by adding these values across the predictors, is employed to predict and forecast the likelihood of vaginal birth (Figure 4).

### Pregnancy outcomes of women in the TOLAC group

The VBAC group exhibited a reduced incidence of intrapartum and postpartum blood loss, a lower rate of puerperal infection, uterine rupture and neonatal asphyxia when compared to the TOLAC-failure group. In comparison with the ERCD group, the VBAC group exhibited a reduced incidence of intrapartum and 24 h postpartum bleeding, a decreased prevalence of puerperal infection, and a reduced rate of uterine rupture. However, the VBAC group demonstrated a higher incidence of placental abruption, premature birth and neonatal admission to the NICU. The higher rate of preterm deliveries in the VBAC group partly explains the higher need for a neonatal intensive care unit Table 4.

**Table 4:** Comparison of pregnancy outcomes between the VBAC group, TOLAC-failure group and ERCS group.

	VBAC (*n* = 839)	TOLAC-failure (*n* = 225)	ERCD (*n* = 400)	P1	P2
Maternal complication					
Blood loss during birth (ml)	247.94 ± 93.45	400.44 ± 202.70	358.63 ± 112.70	0.000	0.000
24 h blood loss (ml)	398.16 ± 198.21	508.24 ± 244.95	494.54 ± 151.41	0.000	0.000
Postpartum fever (%)	16 (1.91)	19 (8.41)	29 (7.25)	0.000	0.000
Rupture of uterus (%)	0 (0.00)	11 (4.87)	6 (1.50)	0.000	0.000
Placental abruption (%)	21 (2.50)	7 (3.10)	1 (0.25)	0.005	0.620
Blood transfusion (%)	16 (1.91)	5 (2.21)	6 (1.50)	0.612	0.770
Neonatal outcomes					
Preterm (%)	123 (14.66)	15 (6.64)	8 (2.00)	0.000	0.001
Asphyxia (%)	14 (1.67)	11 (4.87)	3 (0.75)	0.194	0.005
Transfer NICU (%)	126 (15.02)	36 (15.93)	27 (6.75)	0.000	0.735

P1: Comparison between VBAC group and TOLAC-failure group.

P2: Comparison between VBAC group and ERCS group.

## Discussion

In this study, we found that maternal age, height, pre-pregnancy BMI, parity, vaginal delivery history, inter-delivery interval, cervical Bishop score, induction methods (oxytocin, balloon catheter, artificial rupture of membranes), natural labor, premature rupture of membranes, and pregnancy complications (hyperthyroidism) were associated with successful TOLAC. Multivariate regression analysis demonstrated that maternal age, height, cervical Bishop score, estimated fetal weight, oxytocin and artificial rupture of the membranes were independent factors influencing the successful of TOLAC. This retrospective analysis revealed that the rate of TOLAC was less than 20%. A survey (23) showed that the TOLAC rate in China was only 1.8%–11.1%. Hospitals in China are generally not enthusiastic about TOLAC. Fear of medical disputes caused by adverse outcomes during trial labour is the biggest obstacle to the promotion of TOLAC. The success rate of vaginal trial labour in our hospital is 79%. Among other researchers, the success rate of TOLAC is 68%–83% (24–26).

Previous studies (1992–2023) have developed 22 prediction models for TOLAC success, with accuracies ranging from 66% to 95.3%. However, most models were based on retrospective data (18/22), and only two underwent internal-external validation (27–30). Although Asian populations accounted for the majority of models (12/22), few specifically targeted Chinese cohorts, and key predictors (e.g., cervical Bishop score, oxytocin use) lacked standardized evaluation (6, 27). Our study addressed these gaps by analyzing 10,277 Chinese women (2017–2023) and developing a nomogram incorporating maternal age, height, cervical Bishop score, and oxytocin use (AUC = 83.1%), outperforming existing Asian models [e.g., Lin et al., AUC = 76.2% (28)]. Notably, we first identified ‘artificial rupture of membranes’ as an independent risk factor for TOLAC failure (OR = 0.593, *p* = 0.010), providing quantitative evidence to guide clinical interventions. Given the low TOLAC uptake in Chinese tertiary hospitals (19.67% in our cohort) and high medico-legal risks (18, 19), this model enables personalized risk stratification, facilitating shared decision-making to reduce unnecessary repeat cesarean deliveries.

Affected by China's two-child policy, the proportion of women aged >35 years has increased significantly. Some studies reported (27, 31, 32) that maternal age may have a negative impact on the success of TOLAC. In this study, the age of the mother was used as an independent predictor of TOLAC and was negatively correlated with the success of TOLAC. A cohort study in Sweden (33) found that that the frequency of uterine rupture was 2.2% in patients aged ≥35 years compared with those aged <35 years, and a Norwegian cohort study (34) found that the risk of uterine rupture during trial of labour was three times higher in women aged ≥35 years than in those aged <35 years among 60,000 women who opted for TOLAC. Furthermore, advanced maternal age has been demonstrated to be associated with an increased risk of pregnancy complications (pre-eclampsia, gestational diabetes, placenta abnormalities) (35), which may result in failure of vaginal birth during TOLAC due to these complications. Clinicians should carefully assess the risks.

Maternal height is not explicitly mentioned in the guidelines (6), but has been shown in some studies to be related to the success of TOLAC (36–38) and the incidence of uterine rupture (34). Height was also analysed in the MFMU (31) cesarean section registry. For every centimetre increase in height, the chance of successful TOLAC increases (OR, 1.06; 95% CI, 1.05–1.07), this study also found that maternal height is associated with an increase in TOLAC success, and that for every 1 cm increase in height, the chance of TOLAC success increases (OR 1.079; 95% CI, 1.040–1.119). Although maternal height is not the only factor considered in clinical decision-making for TOLAC, clinicians may consider this factor when counselling patients about the success rate and risks of TOLAC.

Cervical Bishop score is a relatively subjective indicator. In this study, it was assessed by midwives and doctors when the patient was admitted to the hospital. Analysis of the results showed that cervical Bishop score was positively correlated with the success of TOLAC and was the strongest predictor of successful vaginal trial (AUC = 0.721, *p* = 0.000), which is similar to other studies (39, 40). The success rate of TOLAC is closely related to the estimated fetal weight. Previous studies have shown that a fetal weight greater than 3.45 kg triples the chance of a cesarean section, and a fetal weight greater than 3.70 kg reduces the chance of a successful TOLAC by 50% (41). Although ultrasound can predict birth weight with an error of 6%–15% (42), fetal weight management is also necessary for women who wish to try vaginal birth.

This study found that induction of labour after cesarean section (oxytocin, artificial rupture of membranes to induce labour) reduces the success rate of TOLAC, which may be related to some complications during the induction process (such as abnormal fetal heart monitoring, placental abruption, and uterine rupture). Cao et al. (43) found that the method of induction of labour can to some extent improve the success rate of vaginal trial after cesarean section and improve the Apgar score of newborns. However, this study has not further explored the impact of induction of labour on the delivery outcomes of pregnant women with TOLAC. Whether to use induction of labour for women who choose TOLAC without natural labour and the specific plan for induction of labour should be fully assessed and decided under the guidance of a professional doctor.

Although the model included variables such as height, which may vary across populations, it is important to note that significant differences in average height and its distribution exist among populations in different countries or regions. For instance, the average height in Northern European populations is typically higher than in Asian populations. Such variations could influence the predictive performance of the model in other populations. Nevertheless, the prediction model established in this study holds methodological universality. The independent predictors identified (e.g., cervical Bishop score, estimated fetal weight, and oxytocin use) have been consistently associated with TOLAC success rates in multiple international studies (27, 28, 39, 40). Furthermore, the statistical methods employed (e.g., multivariate logistic regression, ROC curve analysis) provide a technical foundation for cross-population validation. To adapt the model to other populations, we recommend parameter calibration or revalidation using local data, such as adjusting height thresholds or incorporating population-specific covariates.

The decision to pursue a TOLAC or opt for an ERCD is multifactorial, involving obstetric, neonatal, and maternal considerations. Among these, maternal preference plays a pivotal role, as it directly reflects the patient's autonomy and informed choice. Wu et al. (44) conducted a longitudinal survey on preferences for delivery methods after a caesarean section and found that the degree of preference for vaginal delivery among women attempting vaginal delivery can predict the final delivery method. Our study of establishing a prediction model could be a clinically important tool as it can be used to identify women with greater chance of a successful TOLAC. Those women with an estimated high probability of successful TOLAC (younger, taller, cervical maturity, lower estimated fetal weight) could be counselled and informed that pursuing a TOLAC is worthwhile since a successful TOLAC is associated with a shorter postpartum recovery time with fewer complications.

### Strengths and limitations

We successfully developed a model to predict the success rate of TOLAC. The main advantages of this study are that its inclusion and exclusion criteria are consistent with clinical guidelines, and the sample size is larger than that of similar studies. It is of some significance for guiding clinicians in our region and for maternal delivery decisions. The shortcomings are that this study is a single-centre retrospective study. To enhance the generalizability of the model, future research should involve multicenter international collaborations, enrolling diverse populations across ethnicities, geographic regions, and socioeconomic backgrounds to evaluate cross-cultural adaptability. Additionally, exploring standardized metrics may help mitigate the impact of population differences on predictive outcomes.

## Conclusions

The fundamental objectives are to improve the success rate of TOLAC, reduce the CS rate, ensure the safety of mother and fetal, and improve long-term prognosis. Our study of establishing a prediction model could be a clinically important tool as it can be used to identify women with greater chance of a successful TOLAC. Those women with an estimated high probability of successful TOLAC (younger, taller, higher cervical Bishop score, lower estimated fetal weight) could be counselled and informed that pursuing a TOLAC is worthwhile since a successful TOLAC is associated with a shorter postpartum recovery time with fewer complications.
